# Ketamine ameliorates hypoxia-induced endothelial injury in human umbilical vein endothelial cells

**DOI:** 10.6061/clinics/2020/e1865

**Published:** 2020-09-03

**Authors:** Xiaohui Zhou, Jing Liu, Siyi Yang, Yanguang Su, Zhipeng Meng, Yuqin Hu

**Affiliations:** I Department of Endocrinology, Huzhou Central Hospital, Affiliated Central Hospital of HuZhou University, 198 Hongqi Road, Huzhou 31300, Zhejiang, People's Republic of China.; II Department of Anesthesia, Huzhou Maternal & Child Health Care Hospital, Huzhou 313000, Zhejiang, China.; III Affiliated Central Hospital, HuZhou University, Emergency Department of Huzhou Central Hospital, 198 Hongqi Road, Huzhou 31300, Zhejiang, People's Republic of China.; IV Anesthesiology Department, Huzhou Central Hospital, Affiliated Central Hospital of HuZhou University, 198 Hongqi Road, Huzhou 31300, Zhejiang, People's Republic of China.

**Keywords:** Hypoxia, Ketamine, p66Shc, Sirt1

## Abstract

**OBJECTIVES:**

Hypoxia leads to endothelial cell inflammation, apoptosis, and damage, which plays an important role in the complications associated with ischemic cardiovascular disease. As an oxidoreductase, p66Shc plays an important role in the regulation of reactive oxygen species (ROS) production and apoptosis. Ketamine is widely used in clinics. This study was designed to assess the potential protective effect of ketamine against hypoxia-induced injury in human umbilical vein endothelial cells (HUVECs). Moreover, we explored the potential mechanism by which ketamine protected against hypoxia-induced endothelial injury.

**METHODS:**

The protective effects of ketamine against hypoxia-induced injury was assessed using cell viability and adhesion assays, quantitative polymerase chain reaction, and western blotting.

**RESULTS:**

Our data showed that hypoxia reduced HUVEC viability, increased the adhesion between HUVECs and monocytes, and upregulated the expression of endothelial adhesion molecules at the protein and mRNA levels. Moreover, hypoxia increased ROS accumulation and upregulated p66Shc expression. Furthermore, hypoxia downregulated sirt1 expression in HUVECs. Alternatively, ketamine was shown to reverse the hypoxia-mediated reduction of cell viability and increase in the adhesion between HUVECs and monocytes, ameliorate hypoxia-induced ROS accumulation, and suppress p66Shc expression. Moreover, EX527, a sirt1 inhibitor, reversed the protective effects of ketamine against the hypoxia-mediated reduction of cell viability and increase in adhesion between HUVECs and monocytes.

**CONCLUSION:**

Ketamine reduces hypoxia-induced p66Shc expression and attenuates ROS accumulation via upregulating sirt1 in HUVECs, thus attenuating hypoxia-induced endothelial cell inflammation and apoptosis.

## INTRODUCTION

Hypoxia is the pathological state of oxygen deficiency in tissues; it plays an important role in the complications of ischemic cardiovascular disease (CVD) (1). Vascular endothelial cells are the primary sensors of organic hypoxia. The damage induced by hypoxia leads to inflammation and ROS production in endothelial cells, resulting in increased permeability (2), adhesion (3), and apoptosis (4).

Reactive oxygen species (ROS) play an important role in hypoxia-induced endothelial injury (5), resulting in endothelial cell apoptosis and necrosis (6). Additionally, exposure to hypoxia increased inflammation-related molecules in endothelial cells (7).

p66Shc, which functions as a redox enzyme, regulates mitochondrial ROS generation and apoptosis (8). It has been reported that p66Shc knockout mice displayed a prolonged lifespan with reduced intracellular levels of oxidants, and increased resistance to oxidative stress-induced apoptosis, which eventually attenuated the incidence of CVDs (9). This indicates that p66Shc may be a novel therapeutic target for the treatment of CVDs by reducing ROS accumulation.

Sirt1, a NAD^+^-dependent deacetylase (10), is associated with downregulating the expression of p66Shc at the protein and mRNA levels (11). In endothelium-specific sirt1-Tg mice, aortic p66Shc expression was decreased (12). Furthermore, activation of sirt1 inhibits oxidative stress and endothelial cell senescence (13).

Ketamine is a N-methyl-D-aspartic acid (NMDA) receptor antagonist; it is mainly used for anesthesia induction and maintenance, and sedation in intensive care units in clinical settings (14). Ketamine is observed to ameliorate brain injury damage and ischemia by attenuating oxidative stress and apoptosis (15). Additionally, hypoxic stress leads to a reduction in sirt1 transcription levels, while ketamine treatment increases the sirt1 transcription levels in the vertebrate brain (16). The present study aims to investigate the protective role ketamine plays against hypoxia-induced injury in human umbilical vein endothelial cells (HUVECs) *in vitro*, and the mechanism thereof.

## MATERIALS AND METHODS

### Cell culture and reagents

First, HUVECs (Clonetics; Lonza, Basel, Switzerland) were cultured in Dulbecco’s modified Eagle medium (DMEM) containing 10% FBS (GIBCO, Australia), 100 U/mL penicillin, and 100 mg/mL streptomycin at 37°C and maintained under 5% CO_2_ conditions. The hypoxia-treated cells were cultured in a modular incubator at 37°C, in an atmosphere of 1% O_2_, 5% CO_2_, and 94% N_2_ (17).

EX527, a sirt1 inhibitor, was purchased from Selleck, China. Ketamine (Sigma-Aldrich, St. Louis, Mo, USA) was dissolved in DMEM. The clinically acceptable concentration of ketamine is approximately 2-80 µM (18,19). Moreover, the half-life time of ketamine is about 2h. Therefore, after hypoxia pretreatment, cells were incubated with different concentrations (1, 2, and 4 μg/mL) of ketamine for 2h. The minimal concentration of ketamine with notable inhibitory effects on the hypoxia-induced reduction of cell viability and increase of monocyte/endothelial cell adhesion was recorded.

### Cell viability assay

HUVECs suspension (3000 cells/100 μL) was added to each well in a 96-multiwell culture plate, followed by incubation at 37°C for 24h. After the corresponding treatments, 10 μL of Cell Counting Kit-8 reagent (CCK-8, Beyotime Institute of Biotechnology, Shanghai, China) was added into each well, followed by incubation for a further 2h. Cell viability was measured using the CCK-8 assay. The optical density of the sample was measured at 450 nm using a microplate reader (BioTek).

### Adhesion assay

The isolation of human peripheral monocytes was acquired with the use of Histopaque-1077 (Sigma). Briefly, 8 mL of heparinized blood from healthy volunteers was layered onto 8 mL of Histopaque-1077. The monocytes were obtained by centrifugation of the blood samples at 400*g* for 30 min. Thereafter, monocytes were washed twice with PBS, and then, the HUVECs subjected to the corresponding treatments were added. HUVECs and monocytes were incubated at 37°C for 30 min, then washed with PBS thrice, and observed using a phase contrast light microscope (AmScope). Adherent monocytes were counted in eight different fields from five samples. Written informed consent was obtained from the volunteers for the publication of this study and any accompanying images.

### Intracellular ROS detection

Intracellular ROS levels were measured using a Reactive Oxygen Species Assay Kit (S0033, Beyotime Co., Shanghai, China) by flow cytometry in accordance with the manufacturer’s instructions. Briefly, cells were seeded in 6-well plates (2×10^6^ cells/mL). Cells were washed with serum-free medium three times after the respective treatments, and then incubated with dichloro-dihydro-fluorescein diacetate (DCFH-DA) for 30 min at 37°C. After being washed three times with serum-free medium, the cells were suspended in 1 mL of ice-cold PBS for flow cytometry analysis, conducted with the cytomics TM FC 500 instrument (Beckman Coulter), with excitation and emission wavelengths of 495 and 525 nm, respectively. The levels of ROS were presented as means±standard deviations (SDs).

### Western blot analysis

Whole-cell protein extracts were obtained by cell lysis buffer (Cell Signaling Technology, Danvers, MA). Same amounts of proteins (60 μg) from HUVECs subjected to different treatments were separated by 8 or 10% SDS-PAGE and transferred to polyvinylidene difluoride (PVDF) membranes at a constant voltage (100 V) for 2h. After culturing in 5% fat-free milk solution, the membranes were cultured with specific primary antibodies at 4°C overnight. The predominant antibodies used were: anti-β-actin monoclonal antibodies, and anti-p66Shc, anti-sirt1, anti-E-selectin, anti-ICAM-1, and anti-active caspase-3 antibodies (Proteintech, Wuhan, P.R. China, 1:1000). Thereafter, primary antibodies were washed with tris-buffered saline containing Tween^®^ 20 and the membranes were incubated with the corresponding secondary antibodies (Proteintech, Wuhan, P.R. China, 1:1000) for 1h at 26°. Subsequently, the membranes were washed, and the specific protein bands were detected using an enhanced chemiluminescence (ECL) system (Milipore, Massachusetts). The respective densities of the protein bands were analyzed using Scan-gel-it software 7.1. In this study, β-actin was used as the loading control.

### Quantitative polymerase chain reaction (qPCR) assay

The relative mRNA expression of ICAM and E-Selectin were detected by quantitative polymerase chain reaction (qPCR). The total RNA was extracted from the cells using Trizol reagent (Invitrogen Life Technologies, Carlsbad, CA, USA) and reverse transcribed to cDNA using RT-qPCR, according to the manufacturer’s instructions. Quantitative real-time fluorescence quantitative PCR was carried out using the SYBR Green qPCR master mix to prepare the PCR reaction mixtures. We designed an internal control (GAPDH) to normalize the relative expression of each target gene using the 2-ΔΔCT (cycle threshold, CT) method, and the relative change of mRNA expression was then measured. Primers sequences used were: ICAM: F 5′-TGCAAGAAGATAGCCAACCAAT-3′, R 5′-GTACACGGTGAGGAAGGTTTTA-3′; E-SELECTIN: F 5′-TGGAACACAACCTGTACATTTG-3′, R 5′-AATTCCCAGATGAGGTACACTG-3′.

The PCR reaction conditions were as follows: initial denaturation at 95° C for 30s, annealing at 56°C for 40s, elongation at 72°C for 40s, and storing at 72°C for 10 min, for a total of 40 cycles.

### Statistical analysis

Data were obtained from five experiments and expressed as means±SDs. N represents the number of times the experiments were repeated using different cell cultures. Statistical significance between conditions was assessed by one-way ANOVA. *p*<0.05 was considered statistically significant.

## RESULTS

### Hypoxia reduced HUVEC viability and increased the adhesion between monocytes/HUVECs, which were reversed by ketamine

**Figure 1:**
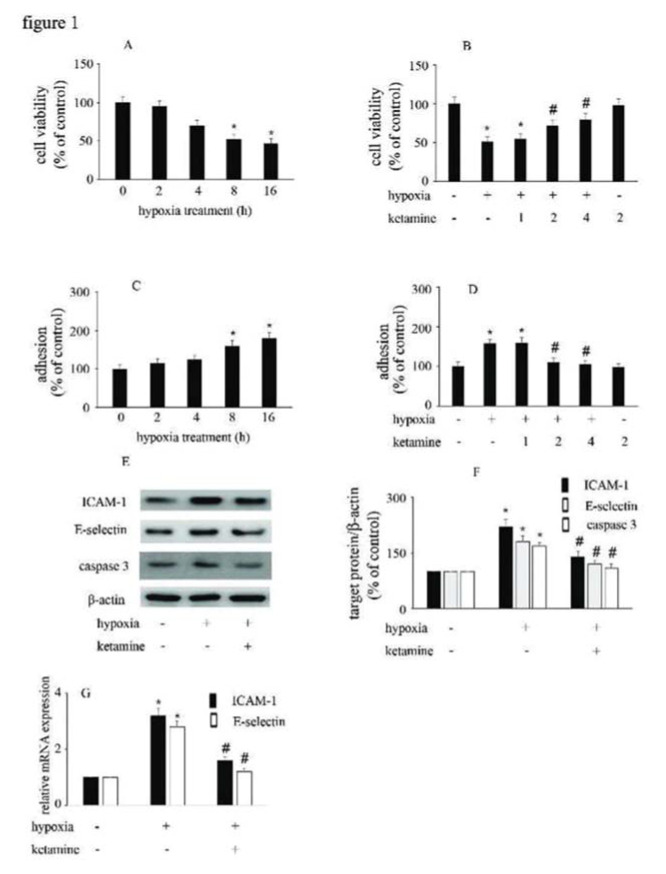
The effect of ketamine on HUVEC viability and monocyte/endothelial cell adhesion following the exposure of HUVECs to hypoxia. (A) Hypoxia reduced the viability of HUVECs in a time-dependent manner. (B) Ketamine enhanced the hypoxia-mediated reduction of cell viability in a concentration-dependent manner. (C) Hypoxia increased monocyte/endothelial cell adhesion in a time-dependent manner. (D) Ketamine inhibited the hypoxia-mediated monocyte/endothelial cell adhesion in a concentration-dependent manner. The optimal concentration of ketamine that began to ameliorate the hypoxia-mediated reduction of HUVEC viability and inhibit the hypoxia-mediated monocyte/endothelial cell adhesion was 2 μg/mL. (E) Equal amounts of proteins were separated by SDS-PAGE and immunoblotted with antibodies against ICAM-1, E-selectin, and caspase-3. (F) The ratio of the protein expression of each specific protein (ICAM-1, E-selectin, and caspase 3) to the expression of β-actin (*p<0.05 *versus* the control group, ^#^*p*<0.05 *versus* the hypoxia group, n=5).

Compared with the control group, hypoxia reduced HUVEC viability and augmented the interactions between monocytes/HUVECs in a time-dependent manner (Figure 1A, C). Moreover, ketamine ameliorated hypoxia-mediated cell injury in a concentration-dependent manner. Compared with hypoxia treatment, 2 μg/mL ketamine was found to reverse hypoxia-mediated reduction in cell viability and increase in monocyte/HUVEC adhesion (Figure 1B, D). This treatment condition was employed in the further analyses to study the potential mechanism responsible for the protective effects of ketamine against hypoxia-mediated endothelial injury.

Compared with the control group, hypoxia enhanced the expression of ICAM-1, E-selectin, and caspase-3. Moreover, ketamine was found to inhibit the hypoxia-induced upregulation of ICAM-1, E-selectin, and caspase-3 expression (Figure 1E-G).

### Hypoxia increased ROS accumulation and p66Shc expression and downregulated sirt1 expression, which were reversed by ketamine

**Figure 2:**
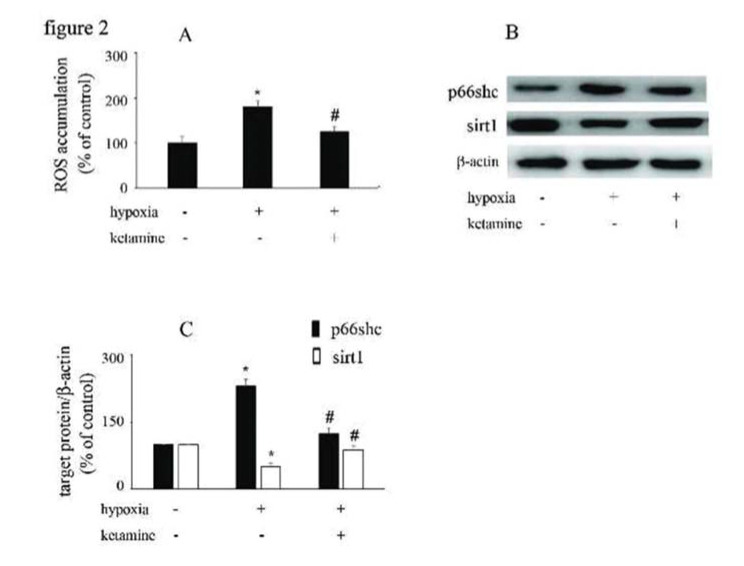
The effects of ketamine on p66Shc and sirt1 expression in hypoxia-treated HUVECs. (A) Hypoxia induced ROS accumulation in HUVECs, which could be reversed by ketamine treatment. (B) Equal amounts of proteins were separated by SDS-PAGE and immunoblotted with antibodies against p66Shc and sirt1. (C) The ratio of the protein expression of each specific protein (p66Shc and sirt1) to the expression of β-actin (*p<0.05 *versus* the control group, ^#^*p*<0.05 *versus* the hypoxia group, n=5).

Compared with the control group, hypoxia increased the accumulation of ROS in HUVECs, which was reversed by ketamine treatment (Figure 2A). Hypoxia increased the expression of p66Shc and decreased the expression of sirt1, while these effects were counteracted by ketamine treatment (Figure 2B, C).

### p66Shc and sirt1 expression and ROS accumulation were modified by hypoxia, ketamine, and EX527 in HUVECs

**Figure 3:**
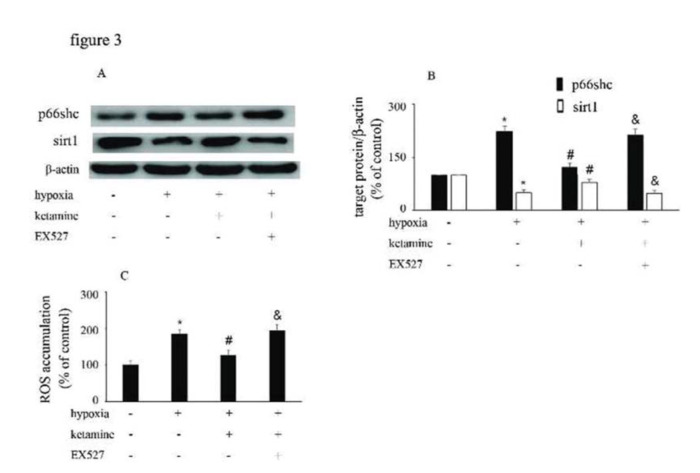
p66Shc and sirt1 expression and ROS accumulation were altered by hypoxia, ketamine, and EX527 in HUVECs. (A) Equal amounts of proteins from HUVECs following the corresponding treatments were separated by SDS-PAGE and immunoblotted with antibodies against p66Shc and sirt1. (B) The ratio of the protein expression of each specific protein (p66Shc and sirt1) to the expression of β-actin. (C) ROS accumulation in HUVECs following the corresponding treatments (*p<0.05 *versus* the control group, ^#^*p*<0.05 *versus* the hypoxia group, & *p*<0.05 *versus* the ketamine group, n=5).

Hypoxia treatment enhanced p66Shc expression, but downregulated sirt1 expression. However, ketamine was shown to reverse the hypoxia-mediated upregulation of p66Shc expression and reduction of sirt1 expression. Furthermore, EX527, a sirt1 inhibitor, was shown to counteract the effects of ketamine (Figure 3A, B). Compared with the control group, hypoxia enhanced ROS accumulation, which was reduced by ketamine. Moreover, EX527 could counteract the effects of ketamine against hypoxia-mediated ROS accumulation (Figure 3C).

### The viability of HUVECs and monocyte/HUVEC adhesion were modified by hypoxia, ketamine, and EX527

**Figure 4:**
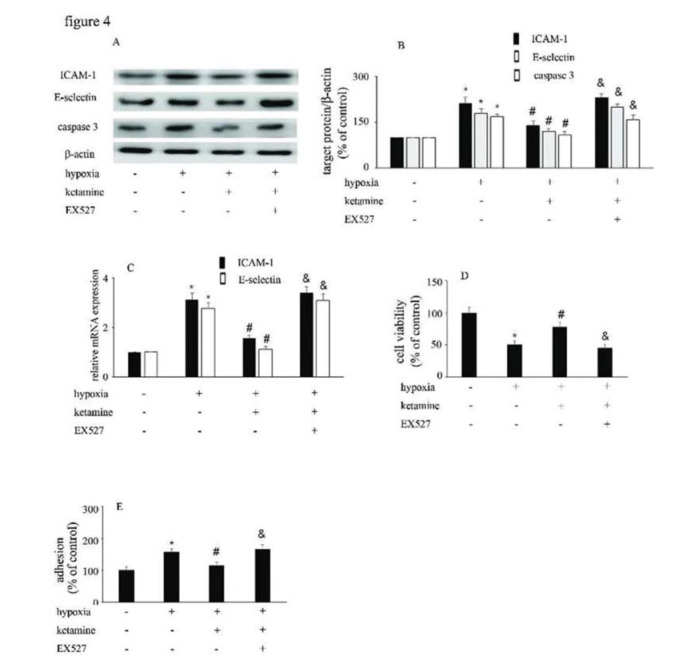
The viability of HUVECs and the HUVEC/monocyte adhesion ability were modified by hypoxia, ketamine, and EX527. (A) Equal amounts of proteins were separated by SDS-PAGE and immunoblotted with antibodies against ICAM-1, E-selectin, and caspase-3. (B) The ratio of the protein expression of each specific protein (ICAM-1, E-selectin, and caspase-3) to the expression of β-actin. (C) Ketamine reversed the hypoxia-mediated decrease of HUVEC viability, while the effect of ketamine was counteracted by EX527. (D) Ketamine reduced the hypoxia-induced increase in monocyte/endothelial cell adhesion, while this effect of ketamine was counteracted by EX527. (*p<0.05 *versus* the control group, ^#^*p*<0.05 *versus* the hypoxia group, & *p*<0.05 *versus* the ketamine group, n=5).

Hypoxia upregulated the expression of ICAM-1 and E-selectin (Figure 4A-C), and augmented the interaction between monocytes/HUVECs (Figure 4E); ketamine treatment was shown to reverse these processes (Figure 4A-C, E). Similarly, hypoxia suppressed the viability of (Figure 4D), and increase caspase-3 expression in, HUVECs; ketamine treatment reversed these effects (Figure 4A, B). Moreover, the effects of ketamine against hypoxia-mediated endothelial injury were counteracted by EX527 treatment (Figure 4).

## DISCUSSION

Hypoxia is a state of insufficient oxygen supply in cells and tissues, and it remains the main reason for the high incidence rate of death and morbidity related to anesthesia (20). Ischemia-induced endothelial injury that occurs after hypoxia results in the increased expression of cell adhesion molecules, such as ICAM-1 and E-selectin, which interact with circulating neutrophils, inducing them to migrate to the sites of endothelium, to further aggravate endothelial cell inflammation and damage (21). Moreover, ROS also play a vital role in hypoxia-mediated endothelial inflammation (22). An increase in ROS accumulation under hypoxic conditions can lead to endothelial cell apoptosis and necrosis (6). The 66-kDa subtype p66Shc of the growth factor adapter shc is involved in ROS generation (23); p66Shc is phosphorylated on Ser36 and translocated to the mitochondria after activation by oxidative stress. In mitochondria, p66Shc combines with cytochromes to produce ROS as an oxidoreductase, which results in apoptosis (24). Sirt1 has been reported to suppress p66Shc transcription via epigenetic chromatin modification through decreased levels of acetylated histone H3, which binds to the promoter region of p66Shc (25). Furthermore, sirt1 was downregulated in the early phase of ischemia, which resulted in increased oxidative stress and apoptosis (26). The inhibition of p66Shc expression is useful to prevent endothelial cell aging and dysfunction caused by oxidative stress. Additionally, accumulation of ROS and expression of p66Shc were positively correlated with the endogenous apoptotic pathway related to oxidative stress (27). Similar results were obtained in the present study, as hypoxia inhibited sirt1 expression and upregulated p66Shc expression, inducing the expression of ICAM-1, E-selectin, and active caspase-3, which increased the interactions between HUVECs/monocytes and endothelial cell apoptosis. Ketamine is an antagonist of the NMDA receptor. It is reported to be a neuroprotective agent that suppresses oxidative stress, cellular dysfunction, and apoptosis (28). Ketamine was found to elicit neuroprotective effects by markedly inhibiting oxidative stress in mice with traumatic brain injury (29). Moreover, a standard clinical dose of ketamine was noted to reduce the inflammatory response in the fetal cerebral cortex following transient hypoxia (30). Additionally, ketamine was found to ameliorate hypoxia-mediated inflammatory and apoptotic pathways in fetal ovine kidneys (31). However, few studies on the anti-oxidative effect of ketamine on vascular endothelial cells exist. Our findings indicated that ketamine could inhibit the hypoxia-mediated interactions between monocytes/endothelial cells and increase of endothelial cell apoptosis. Moreover, ketamine decreased hypoxia-induced ROS accumulation and p66Shc expression via the upregulation of sirt1 expression.

This study has a few limitations. First, we performed all experiments *in vitro*, and *in vivo* experiments are required to verify our hypothesis. Furthermore, we proposed that ketamine inhibits the hypoxia-induced increase in p66Shc expression via upregulating sirt1 expression in HUVECs. However, knockdown and overexpression of sirt1 should be further researched to provide more persuasive conclusions.

In summary, the present study indicated that ketamine reduced the hypoxia-induced upregulation of p66Shc expression and ROS accumulation via upregulating sirt1 expression in HUVECs, thus ameliorating the hypoxia-mediated reduction in HUVEC viability and reducing monocyte/endothelial cell adhesion.

## AUTHOR CONTRIBUTIONS

Meng Z and Hu Y designed the study, supervised the experiments, analyzed the data and wrote the manuscript. Zhou X and Liu J performed the experiments. Yang S and Su Y collected the data.
